# Childhood ADHD and Its Adult Mimics: When First-Time ADHD Diagnoses in Adulthood May Reflect Other Conditions—A Narrative Review

**DOI:** 10.3390/brainsci16090965

**Published:** 2026-09-13

**Authors:** Alessandro Cuomo, Mario Pinzi, Andrea Fagiolini

**Affiliations:** Department of Molecular Medicine, Division of Psychiatry, University of Siena, Viale Bracci 16, 53100 Siena, Italy; alessandro.cuomo@unisi.it (A.C.);

**Keywords:** ADHD across the lifespan, childhood and adolescent ADHD, late-onset ADHD, diagnostic inflation, developmental trajectory, executive function, borderline personality disorder, stimulant diversion, retrospective diagnosis, differential diagnosis, comorbidity

## Abstract

**Highlights:**

**What are the main findings?**

- ADHD changes across development, shifting from overt hyperactivity and impulsivity in childhood toward inner restlessness, executive dysfunction, disorganization, and impaired self-regulation in adulthood.
- The recent increase in adult ADHD diagnoses is compatible with several overlapping processes, including improved recognition of historically missed cases, broadened criteria, expanded access to assessment, and possible over-attribution in a subset of adult presentations; current evidence does not quantify their relative contributions.
- In some cases, a first-time adult ADHD diagnosis may instead reflect an alternative or co-occurring mood, anxiety, substance-use, sleep, stress-related, or personality condition; longitudinal studies also document associations between childhood ADHD and later borderline personality disorder or bipolar disorder without establishing diagnostic substitution.

**What are the implications of the main findings?**

- Adult ADHD assessment should combine a developmentally informed history with collateral information and systematic evaluation of mood, anxiety, substance-use, sleep, stress-related, and personality disorders, rather than relying on screening questionnaires alone.
- Apparent late-onset ADHD and retrospective rediagnosis should be approached cautiously, explicitly acknowledging diagnostic uncertainty and distinguishing clinical diagnosis from occupational, administrative, forensic, or medication-related considerations.
- Longitudinal associations between childhood ADHD and later personality or mood pathology strengthen the rationale for early identification and evidence-based treatment, but do not establish that early treatment prevents those later outcomes.

**Abstract:**

**Background/Objectives:** Attention-deficit/hyperactivity disorder (ADHD) begins in childhood, but its presentation changes over time. Adult ADHD is increasingly recognized; in the United States, 2023 survey data indicate that approximately 6% of adults reported a current diagnosis. This narrative review examines the developmental course of ADHD from childhood to adulthood and the controversial phenomenon of apparent late-onset presentations. Broadened diagnostic criteria, greater public awareness, and expanded access through telehealth may contribute to the rise in adult diagnoses, but these factors do not establish population-level diagnostic inflation. In some first-time adult assessments, the presenting symptoms may be better explained by an alternative or co-occurring condition; longitudinal studies also document associations between childhood ADHD and later borderline personality disorder or bipolar disorder without showing that diagnostic substitution is common. **Methods:** This is a narrative, non-systematic review. The primary and secondary literature were identified through targeted searches of PubMed/MEDLINE and Google Scholar (studies published through July 2026) using combinations of the terms “adult ADHD”, “diagnostic inflation”, “late-onset ADHD”, “executive function”, “borderline personality disorder”, “retrospective recall” and “stimulant diversion”, supplemented by hand-searching the reference lists of key reviews, current diagnostic manuals (DSM-5, DSM-5-TR, ICD-11) and consensus statements. All cited empirical claims and figures were verified against the primary source before submission. **Results:** The clinical presentation of ADHD shifts from overt hyperactive–impulsive behaviour in childhood toward executive dysfunction and subjective restlessness in adulthood. Longitudinal cohorts show both persistence and fluctuating remission, while birth-cohort evidence for apparently late-onset ADHD is substantially reduced when cases undergo repeated, multi-informant reassessment. Evidence for the recent rise in adult diagnoses is most direct for criterial broadening and expanded access, is qualitatively strong but difficult to quantify for correction of historical underrecognition and remains contested for genuinely adult-onset ADHD. Mood, anxiety, substance-use, sleep, stress-related, and personality conditions can overlap with or mimic ADHD-like symptoms. Prospective studies also report associations between childhood ADHD and later borderline personality disorder or bipolar disorder, but do not establish how often adult ADHD is diagnosed instead of either condition. Screening instruments and retrospective childhood recall therefore require interpretation within a full developmental and differential assessment. **Conclusions:** Adult ADHD is a genuine, historically underrecognized, and treatable condition. Its increased visibility warrants careful assessment, because a later diagnosis based on weakly corroborated retrospective evidence may be overconfident even when it is ultimately correct. Maintaining diagnostic integrity across the lifespan requires developmentally informed assessment, systematic differential diagnosis, cautious use of screening instruments, and explicit separation of clinical conclusions from administrative or medico-legal determinations.

## 1. Introduction

For most of the twentieth century, attention-deficit/hyperactivity disorder (ADHD) was treated almost exclusively as a disorder of childhood, one that clinicians expected patients to outgrow by adolescence. That assumption has been overturned. A substantial body of longitudinal research, following children diagnosed with the disorder into their thirties and forties, has demonstrated that although overt hyperactivity often subsides, difficulties with sustained attention, organization, impulse control and emotional regulation frequently continue into adult life [1,2]. This finding reshaped clinical practice: adult ADHD is now assessed, treated and researched as a distinct area of psychiatry in its own right.

The recognition of adult ADHD has brought substantial benefits. Many adults who spent years attributing chronic underachievement, unstable employment, or relationship strain to personal failing have gained access to an explanatory formulation and to treatments that can improve functioning [3]. At the same time, increasing recognition creates a need to distinguish historically missed ADHD from nonspecific attentional complaints and from symptoms better explained by another condition. This review examines that diagnostic task without treating the rise in diagnoses itself as evidence that false-positive diagnoses have increased.

For the purposes of this review, diagnostic inflation means an increase in adult ADHD diagnoses that is not accounted for by genuine historical underrecognition, expanded access to evaluation, or legitimate criterial broadening, but instead reflects a lowered evidentiary threshold: reliance on a screening score in place of full evaluation, an insufficiently corroborated retrospective narrative, or symptom attribution that overlooks a better-fitting alternative diagnosis. This is conceptually distinct from increased detection of previously missed cases, diagnostic substitution, and criterial broadening. Available population-level evidence does not separate or quantify the contributions of these processes; diagnostic inflation is therefore treated here as a clinically relevant hypothesis rather than an established population-level phenomenon [4,5,6,7,8,9,10]. Framed this way, the review asks how ADHD presentation changes across development; how persistence and apparent late onset are distinguished; how diagnostic criteria affect eligibility; which conditions overlap with adult ADHD; how self-report and retrospective recall should be interpreted; and which safeguards are supported in clinical, occupational, and forensic assessment. Section 6 considers criterial broadening, historical underrecognition, healthcare access, and apparent adult-onset ADHD separately rather than treating the rise in diagnoses as a single process.

The article follows that structure across the developmental arc. It reviews childhood, adolescent, and adult presentations; persistence and apparent late onset; changes in diagnostic criteria; neurobiological and genetic models; and the overlap of ADHD symptoms with mood, anxiety, substance-use, sleep, stress-related, and personality conditions. Longitudinal associations between childhood ADHD and later borderline personality disorder or bipolar disorder are considered separately from comorbidity and diagnostic substitution. The review then addresses screening instruments, retrospective childhood recall, and clinical, occupational, administrative, and forensic considerations. Autism-spectrum and other neurodevelopmental conditions are noted where relevant to differential assessment but are not reviewed comprehensively.

## 2. Materials and Methods

This is a narrative review rather than a systematic review or meta-analysis, reflecting the paper’s aim of synthesising developmental, diagnostic, neurobiological and medico-legal perspectives on adult ADHD into a clinically oriented narrative synthesis, rather than of quantifying a single pooled effect. The literature was identified through targeted searches of PubMed/MEDLINE and Google Scholar, covering material published through July 2026, using combinations of the terms “adult ADHD”, “late-onset ADHD”, “diagnostic inflation”, “ADHD persistence”, “executive function”, “borderline personality disorder”, “retrospective recall”, “ADHD self-report scale” and “stimulant diversion”. Reference lists of major reviews, consensus statements and the current diagnostic manuals (DSM-5, DSM-5-TR, ICD-11) were hand-searched for additional sources. Priority was given to prospective longitudinal cohort studies, large-scale epidemiological surveys, systematic reviews and meta-analyses, and consensus or guideline documents from recognized professional bodies; case reports and non-peer-reviewed sources were excluded. The literature on the recent rise in adult diagnoses was further organized into four candidate contributing factors, diagnostic criteria broadening, increased detection of historically underrecognized presentations, expanded healthcare access, and apparent adult-onset ADHD (Section 6), so that the strength of evidence for each could be assessed separately rather than treated as a single undifferentiated trend. Because this is a narrative rather than a systematic review, the literature was drawn primarily from PubMed/MEDLINE and Google Scholar rather than a multi-database protocol, search terms and dates are reported as a general strategy rather than a fully reproducible search string, and no formal risk-of-bias appraisal or independent dual screening was performed; these choices reflect the paper’s aim of building a clinically oriented synthesis rather than a comprehensive or reproducible evidence map, and are discussed further as limitations in Section 12. To reduce one-sided interpretation, the review presents evidence for genuine historical underrecognition alongside evidence relevant to possible overextension, identifies countervailing findings, and labels areas of uncertainty explicitly; the conclusions should nevertheless be read in light of the purposive, non-systematic study selection described above. Every empirical claim, statistic and attribution cited in this review was independently checked against the original primary source at the time of writing to minimise citation drift. As a narrative synthesis, this review does not follow a PRISMA protocol, was not pre-registered, and does not report formal risk-of-bias ratings for included studies; conclusions should be read as an informed clinical synthesis rather than as a quantitative summary effect. The authors used Claude (Anthropic) for English-language editing, creating the graphic abstract, initial reference formatting, and document formatting; AI-suggested reference details (journal, year, volume) were independently checked by the authors against the original publication or its indexed record before submission, and inaccuracies identified in an earlier draft were corrected accordingly. The authors critically reviewed all AI-generated outputs, edited the final manuscript, and verified every statement and reference against its primary source. The authors take full responsibility for the submitted content, including for any residual error.

## 3. Diagnostic Visibility and Evidentiary Standards in Retrospective Reassessment

Adult ADHD has become substantially more visible in clinical services and public discourse. In a 2023 United States survey, approximately 6% of adults reported a current ADHD diagnosis, and about half of those diagnoses had first been made in adulthood [5]. At the same time, historical underrecognition remains well-documented, particularly among women and people with predominantly inattentive presentations [3,11]. These findings create two simultaneous clinical obligations: not to dismiss plausible previously missed ADHD, and not to treat recent diagnostic visibility or a positive screening score as sufficient evidence of childhood-onset ADHD. Guidelines and empirically informed recommendations instead require a full developmental and psychiatric assessment, evaluation across settings, and consideration of alternative explanations [3,10,12].

Retrospective reassessment is intrinsically difficult because adult recall of childhood ADHD has limited predictive accuracy when compared with contemporaneous childhood information, and estimated persistence varies substantially with the informant and diagnostic method used [7,8,13]. A later clinician may reasonably revise an earlier formulation, but a current diagnosis alone cannot establish that the earlier assessment was mistaken or negligent. A self-report screener and an uncorroborated retrospective account should therefore initiate, not replace, a fuller evaluation [6,10].

Accordingly, this review treats retrospective rediagnosis as an evidentiary question rather than as a question of clinician or patient motivation. Its credibility depends on reconstructing developmental onset and impairment, seeking collateral information where available, applying current diagnostic criteria, and testing competing or co-occurring explanations [3,4,10,12]. The same standard applies when borderline personality disorder is considered, because symptom overlap and genuine comorbidity are both documented [14,15,16].

## 4. From a Childhood Syndrome to a Lifespan Condition

The clinical description of what is now called ADHD has passed through several names, including minimal brain dysfunction, hyperkinetic reaction of childhood, and attention deficit disorder with and without hyperactivity, before settling into its current form across DSM-III, DSM-IV and DSM-5 [17]. Each relabeling reflected a shift in emphasis: from presumed neurological damage, to behavioral hyperactivity, to a more balanced view centered on inattention and executive dysfunction. Throughout most of this history, the diagnosis was anchored almost entirely in school-age behavior, and adult presentations, when they were noticed at all, were folded into mood, anxiety or personality frameworks [2,3,18].

The turn toward adulthood began in earnest in the 1990s and accelerated through the 2000s, as prospective cohort studies of hyperactive children reached adulthood and were systematically reassessed. These studies converged on a consistent picture: a meaningful proportion of children with ADHD (estimates commonly range from a third to two-thirds, depending on the definition of persistence used) continue to meet full or partial criteria as adults [2]. Meta-analytic and longitudinal work further indicates that this persistence is not fixed: the probability of continued impairment declines gradually with age even among those who initially qualify [19], and course over time is itself variable, with many individuals cycling between fuller and more partial symptom presentations rather than following a single trajectory of persistence or remission [20]. Epidemiological surveys subsequently estimated the prevalence of current adult ADHD in the United States at approximately 4.4% of adults aged 18–44 [21], while cross-national and more recent meta-analytic estimates converge on a somewhat lower figure, generally in the region of 2.5–3% of the adult general population once methodological heterogeneity across studies is accounted for [22,23]. Either figure is high enough to establish the condition as a mainstream concern for adult psychiatry rather than a residual curiosity of child psychiatry.

## 5. Developmental Phenomenology: How ADHD Looks in Childhood, Adolescence and Adulthood

A clinician who expects adult ADHD to resemble childhood ADHD is likely to miss it, because their typical presentations can differ substantially. In early and middle childhood the disorder is usually easiest to recognize in its hyperactive–impulsive form: running or climbing in situations where it is inappropriate, difficulty remaining seated, blurting out answers, interrupting others, and a level of physical restlessness that teachers and parents notice without needing a checklist. Inattentive symptoms are present from the same age but are less visually striking, and are more likely to be overlooked in a child who is quiet rather than disruptive, particularly if the child is also compliant and well-behaved in other respects [2,3,4,11,17].

Through adolescence, overt hyperactivity typically diminishes even when the underlying difficulty persists. What often remains is a subjective sense of inner restlessness rather than visible motor activity, together with mounting difficulty in domains that place increasing demands on independent self-management: keeping track of multiple assignments, planning across longer time horizons, resisting immediate distraction in favor of deferred goals, and regulating frustration in the face of repeated setbacks. Because adolescence is also the developmental period in which parental and school scaffolding is deliberately withdrawn in favor of self-directed study and independent responsibility, symptoms that were previously absorbed by external structure can become newly visible, not because the underlying condition has changed but because the environment has stopped compensating for it [2,3,19,20].

By adulthood, the hyperactive–impulsive presentation described in early diagnostic criteria has usually receded further still, and the clinical picture is dominated by executive dysfunction: chronic disorganization, procrastination on effortful tasks, difficulty initiating or completing multi-step projects, forgetfulness in daily responsibilities, and impulsive decisions in spending, relationships or employment. This is precisely why DSM-5 supplemented its adult criteria with age-appropriate examples, reframing hyperactivity as a subjective sense of restlessness rather than literal climbing or running [4]. The practical implication is significant for diagnosis at every age: a developmental history that asks only whether a patient was ‘hyperactive as a child’ in the literal sense risks missing inattentive-predominant children, quiet adolescents whose difficulties surfaced only once structure was removed, and adults whose only available window onto childhood is a recollection of a period that, in hindsight, looked nothing like the adult picture in front of the clinician (Table 1) [2,3,18,19,20].

## 6. Factors Contributing to the Rise in Adult ADHD Diagnoses

To avoid treating the recent rise in adult ADHD diagnoses as a unitary phenomenon, this section organizes the relevant literature into four candidate contributors: diagnostic-criteria broadening, increased detection of historically underrecognized presentations, expanded healthcare access, and apparent adult-onset ADHD [4,5,9,11,28]. These processes rest on different kinds of evidence and are not equally well-supported; the closing synthesis weighs them separately rather than assuming that any one process explains the observed trend.

### 6.1. Diagnostic Criteria Broadening: What DSM-5 Actually Changed

The revision of ADHD criteria in DSM-5 was designed explicitly to make adult presentations easier to recognize, and it is worth being precise about what changed, because the change itself partly explains the rise in adult diagnoses that followed it. First, the age by which several symptoms must have been present was raised from seven to twelve years, acknowledging that many adults with clinically significant ADHD cannot reliably recall or document impairment before age seven. Second, the number of symptoms required for a diagnosis in individuals aged seventeen and older was lowered from six to five in either the inattentive or hyperactive–impulsive domain. Third, the criteria were supplemented with examples of how symptoms typically present in adolescents and adults, for instance framing hyperactivity as inner restlessness rather than running or climbing [4].

DSM-5-TR, published in 2022, left this structure intact; its contribution was textual refinement rather than a further reorganization of the adult criteria [29]. The World Health Organization’s ICD-11 similarly recognizes ADHD as a condition that can be diagnosed for the first time in adulthood, provided a developmental onset can be established [30]. Together, these changes broadened adult eligibility. Lowering a symptom threshold and relaxing a strict age cutoff would be expected to increase the number of adults who can qualify for the diagnosis, independent of any change in the true prevalence of the underlying condition. One field trial applying both criteria sets within the same representative sample of young adults found a modest but measurable increase in prevalence under DSM-5 relative to DSM-IV (3.55% vs. 2.8%), consistent with a real but not dramatic criterial effect [9]; comparable head-to-head data in older adult samples, and across the full range of clinical settings this review is concerned with, remain limited, so the size of this effect relative to genuine underrecognition remains only partly quantified. Reviews of ADHD prevalence estimates have repeatedly shown that diagnostic criteria and the method of ascertainment, rather than incidence alone, account for much of the variability across studies and across time [22,23]. Any account of the rise in adult ADHD diagnoses must therefore separate three distinct phenomena: a real historical underrecognition of persistent symptoms, a criterial broadening that expands eligibility, and a possible, currently unquantified, lowering of the threshold at which the diagnosis is entertained.

### 6.2. Increased Detection and Historical Underrecognition

One well-documented source of increased detection is the historical underrecognition of women. A systematic review by Attoe and Climie concluded that adult women with ADHD are disproportionately likely to have been missed in childhood, often because their presentation leans toward inattentiveness rather than overt hyperactivity, and because inattentive symptoms are more easily absorbed into everyday expectations of a quiet, conscientious girl [11]. Many women who are eventually diagnosed in adulthood describe a long prior history of chronic disorganization, underachievement relative to evident ability, and exhaustion from compensatory effort, patterns that were previously more likely to be labeled as anxiety, low self-esteem or simple personality traits than investigated as ADHD. This account, framed around presentation style, is necessarily simplified; referral bias, hormonal and developmental factors, and the intersection of gender with race, culture and socioeconomic status all plausibly shape whose difficulties are recognized as ADHD, and a fuller treatment of sex, gender and intersectional factors in ADHD recognition is beyond the scope of this article.

Evidence of historical underrecognition does not mean that nonspecific compensatory strategies establish ADHD retrospectively. List-making, rigid routines, reliance on caffeine, or late-night catch-up work can occur for many reasons and should be interpreted only within a full assessment of developmental onset, cross-situational impairment, and alternative explanations [3,4,10,12]. The clinical task is to recognize genuinely missed ADHD without treating an uncorroborated retrospective narrative as conclusive.

### 6.3. Expanded Healthcare Access and Telehealth

A 2024 analysis from the U.S. National Center for Health Statistics Rapid Surveys System estimated that 15.5 million adults, approximately 6.0% of the adult population, reported a current ADHD diagnosis in 2023, and roughly half had first been diagnosed at age eighteen or older [5]. Approximately one third reported taking stimulant medication, and about half had used telehealth for ADHD-related care [5]. These cross-sectional survey data document extensive telehealth use among adults reporting ADHD but do not determine whether telehealth increased diagnosis or altered diagnostic accuracy. A study of adults self-referred for online ADHD assessment found that an asynchronous self-report tool was more conservative than a virtual clinical interview in assigning a positive diagnosis [31]; however, that comparison concerns a specific online tool and cannot establish diagnostic accuracy across telehealth modalities. The reviewed evidence therefore supports treating telehealth as an access context, not as evidence of diagnostic inflation or reduced assessment quality.

Self-reported diagnostic prevalence should be interpreted as reflecting underlying prevalence together with help-seeking, access, recall, and diagnostic ascertainment. The survey cannot determine which of these factors explains the observed rate or whether any individual diagnosis is incorrect [5,9,10].

### 6.4. Apparent Adult-Onset ADHD: Genuine Late Emergence or Diagnostic Artefact?

A further complication, directly relevant to a diagnosis defined as neurodevelopmental in origin, comes from a cluster of prospective birth-cohort studies published in the mid-2010s. Following participants from early childhood into their twenties with repeated, structured assessments, these studies unexpectedly found that a substantial proportion of young adults who met full criteria for ADHD had little or no documented impairment earlier in childhood. Moffitt and colleagues, using New Zealand’s Dunedin cohort, reported that most young adults meeting ADHD criteria had not met criteria as children, and that the reverse was also true: many children with well-documented ADHD did not meet criteria as adults [32]. Agnew-Blais and colleagues, using the E-Risk twin cohort in the United Kingdom, and Caye and colleagues, using a Brazilian birth cohort, reported closely similar patterns, each identifying a group of young adults with clinically significant ADHD symptoms and impairment but no clear childhood diagnostic history [33,34].

These findings triggered genuine debate rather than settled consensus, and the disagreement matters for how such cases should be handled clinically. One reading takes the findings at face value and proposes that ADHD, or at least a clinically significant attentional syndrome resembling it, can in some individuals first become impairing in young adulthood, perhaps under the pressure of novel demands for independent self-regulation that have no equivalent in a structured childhood. A second, more cautious reading argues that most of these apparent late-onset cases reflect subthreshold childhood symptoms that were real but insufficiently impairing to cross a diagnostic threshold at the time, later becoming clinically significant once parental and school scaffolding was withdrawn, consistent with the same developmental logic described in the previous section. A third reading emphasizes measurement artifact: single-informant assessment, changes in who is reporting symptoms, over-reliance on parent report in childhood, and the confounding effects of emerging substance use, mood or anxiety symptoms in early adulthood, all of which can inflate apparent new-onset caseness without any true change in underlying neurodevelopmental status. Subsequent reassessments that more strictly exclude substance-related, episodic, or informant-discrepant presentations have narrowed the estimated proportion of plausible late-onset cases [28,35]. One influential detailed reassessment is summarised immediately below; a comprehensive systematic synthesis of the broader reassessment literature, beyond the single research programme summarised here, remains a priority for future work.

This debate has since been tested directly, using a more intensive ascertainment strategy than the single-timepoint self-report on which the birth-cohort studies above relied. Sibley and colleagues, drawing on the Multimodal Treatment Study of ADHD (MTA) cohort, applied comprehensive, repeated diagnostic assessments between ages 10 and 25 to every participant who initially appeared to meet criteria for late-onset ADHD [28]. On this closer reassessment, the substantial majority of apparent late-onset cases did not hold up: most were traced to an unreported childhood history, a co-occurring condition such as substance use or another psychiatric disorder, or symptom fluctuation that did not represent a stable new disorder, and only a small minority of the participants initially flagged, on the order of one in twenty, remained plausible cases of genuine late onset after full evaluation. A related qualitative analysis by the same research group found that clinicians who examined these presentations in depth typically identified a specific alternative explanation once collateral information and a detailed history were obtained [35].

A distinct possibility is that genuine childhood ADHD is followed by a different condition that becomes clinically prominent by adulthood. In a prospective longitudinal study of girls with and without childhood ADHD, a meaningful minority of those with childhood ADHD later met criteria for borderline personality disorder, with executive-function deficits and cumulative adversity among the predictors [36]. A systematic review and meta-analysis likewise found an increased later risk of bipolar disorder among individuals with ADHD, although the evidence was heterogeneous and does not establish a single causal developmental pathway [37]. These findings support careful assessment of which condition best explains the current adult presentation; they do not establish that diagnostic substitution is frequent, or that early ADHD treatment prevents later personality or mood disorders. The preventive implication is therefore a hypothesis for prospective study rather than a conclusion of this review.

The detailed reassessment findings support a cautious clinical approach to apparent late-onset ADHD [10,28,35]. A young adult with an impairing attentional syndrome but a thin childhood record deserves a structured evaluation rather than a reflexive verdict in either direction. DSM-5 does not require documentation before age seven, but it does require evidence that several symptoms were present before age twelve [4]. Subthreshold childhood traits, later withdrawal of environmental scaffolding, co-occurring psychiatric or substance-related conditions, and the limitations of delayed recall should all be weighed explicitly [7,10,13,28].

Weighing these four factors separately, direct evidence supports a contribution from criterial broadening, including a modest DSM-5 versus DSM-IV prevalence difference in a representative young-adult sample [4,9]. National survey data document extensive telehealth use among adults reporting ADHD, which is consistent with expanded access but does not establish a causal effect on diagnosis [5]. Historical underrecognition, particularly among women, is well supported but difficult to quantify separately from other drivers of rising diagnosis [11]. Evidence for genuinely adult-onset ADHD remains contested: birth-cohort findings identify apparent cases [32,33,34], whereas detailed repeated reassessment excludes most initially screen-positive cases after accounting for impairment, psychiatric history, substance use, and collateral information [28,35]. These processes are not mutually exclusive, but the reviewed evidence does not quantify their relative population-level contributions [5,9,10,28].

## 7. Neurobiological, Executive-Function and Genetic Models

Neurobiological and genetic findings support ADHD as a valid neurodevelopmental construct but do not diagnose an individual or resolve differential diagnosis. Barkley’s influential model frames ADHD primarily as a disorder of behavioural inhibition, linking weak inhibitory control to secondary impairments in working memory, affect regulation, internalized speech, and planning toward delayed goals [38]. Neuroimaging and molecular studies add evidence of altered dopaminergic and noradrenergic signalling, particularly in striatal and prefrontal circuits involved in reward processing and sustained attention [39].

Genome-wide association studies have added a further, converging line of evidence. A large-scale meta-analysis identified 27 genome-wide significant loci associated with ADHD and highlighted dozens of candidate risk genes enriched among genes expressed in early brain development and in midbrain dopaminergic neurons; the same analysis estimated that a large majority of the common genetic variance influencing ADHD is shared with other psychiatric disorders, including depression and substance use disorders [40]. This genetic overlap is clinically relevant precisely because it complicates, rather than resolves, differential diagnosis: a shared genetic liability across ADHD, mood, and substance-use phenotypes is consistent with genuine comorbidity, but it also means that genetic architecture alone cannot be used to adjudicate which condition best explains a given adult’s presentation.

These findings have two clinical implications. First, stimulant medications affect dopamine and norepinephrine signalling and can rapidly improve core ADHD symptoms, but treatment response is not diagnostically specific and should not be used to establish or retrospectively validate ADHD. Second, group-level neurobiological findings do not determine an individual’s diagnosis; clinical assessment still depends on developmental history, impairment across settings, and systematic evaluation of alternative and co-occurring conditions [3,12,18,38].

## 8. Differential Diagnosis in Adult Practice

Inattention, impulsivity, and emotional reactivity are transdiagnostic features. Adult assessment must therefore determine which process best accounts for the symptoms across the person’s developmental history, current clinical state, and functional contexts [3,14,25,27].

### 8.1. Mood Disorders

Depressive episodes routinely produce impaired concentration, indecisiveness and reduced follow-through, symptoms that overlap heavily with the inattentive presentation of ADHD. The distinguishing feature is usually temporal course: depressive cognitive impairment tends to wax and wane with mood episodes and to be accompanied by anhedonia, sleep and appetite change, and diminished self-worth, whereas ADHD-related inattention is comparatively stable across mood states and traceable to childhood. Bipolar-spectrum presentations raise a related but distinct problem. Racing thoughts, distractibility and impulsive decision-making during hypomanic or mixed episodes can closely resemble ADHD, and the European Consensus Statement on adult ADHD explicitly identifies bipolar disorder as one of the most important conditions to exclude, or to identify as a comorbidity, before treating presumed ADHD with stimulant medication [3], and reviews of comorbid presentations recommend a dimensional approach that assesses mood, anxiety and attentional symptoms together rather than assuming a single diagnosis explains the full clinical picture [27]. The key discriminators are episodicity and diurnal or seasonal patterning: ADHD symptoms are traits present since childhood and largely context-independent, while bipolar symptoms cluster into discrete periods of altered mood, energy and sleep need.

### 8.2. Anxiety Disorders

Chronic anxiety produces its own form of attentional disruption: worry consumes working memory capacity, and hypervigilance fragments sustained attention on tasks unrelated to the perceived threat. Adults with generalized anxiety disorder frequently describe themselves as unable to concentrate, easily overwhelmed and behind on responsibilities, a self-description that overlaps substantially with items on standard ADHD screening scales; psychometric work directly testing this overlap has found that the Adult ADHD Self-Report Scale does not cleanly separate ADHD from anxiety-driven inattention in clinically anxious samples [26], and comorbid anxiety and depression are themselves common in adult ADHD, further complicating single-diagnosis attributions [25]. The distinguishing question is functional: is the attentional lapse driven by intrusive worry about specific concerns, resolving when the worry is addressed, or is it a comparatively content-free difficulty in sustaining attention on any demanding task, present since childhood and largely independent of current stressors?

### 8.3. Substance Use and ADHD

ADHD and substance-use disorders can co-occur and require specific attention in adult assessment. Longitudinal follow-up of children with ADHD has found an elevated later risk of substance-use disorder compared with matched controls, without establishing a simple causal pathway [41]. Alcohol, cannabis, stimulant use, and withdrawal can also produce attentional, memory, sleep, or restlessness symptoms that overlap with ADHD. A substance-use history is therefore a standard component of assessment, and a period of sustained abstinence may help clarify the presentation when clinically feasible [3,12]. Prescribed stimulants have recognized misuse and diversion potential; review evidence supports assessing risk, particularly in adolescent and young-adult populations, without presuming misuse in most patients [42].

### 8.4. Borderline Personality Disorder: Overlapping Presentations and Diagnostic Caution

Borderline personality disorder merits consideration because it can share impulsivity, emotional instability, and interpersonal disruption with ADHD, while its diagnostic formulation emphasizes pervasive instability of affect, self-image, and relationships [43,44]. Empirical studies have documented elevated rates of ADHD symptoms and diagnoses among people with borderline personality disorder [14,15,16,45], supporting careful assessment of both overlap and genuine comorbidity.

The phenomenological distinction, however, remains important. Impulsivity in borderline personality disorder tends to occur in the context of intense, rapidly shifting affective states and unstable relationships, and may serve an interpersonal or self-regulatory function. Impulsivity in ADHD is more consistently associated with impaired inhibitory control and sustained attention across contexts. When ADHD is considered retrospectively in an adult without clear evidence of childhood onset, symptom overlap creates a possibility that emotional dysregulation associated with borderline psychopathology may be misattributed to ADHD. The distinction should therefore be made through developmental, interpersonal, and collateral evidence rather than symptom overlap alone [14,15,16,43].

Borderline personality disorder is heterogeneous, and its symptom overlap with ADHD can make differential diagnosis difficult [14,15,16,43]. A positive ADHD screen in a patient with borderline pathology should therefore be evaluated using the same developmental, functional, and collateral standards applied in every adult ADHD assessment, not a higher or lower standard. Genuine comorbidity is well-documented [14,15,16,45]. The literature reviewed here does not establish how often adult ADHD is diagnosed in place of, rather than alongside, a better-fitting borderline personality disorder formulation; diagnostic substitution, comorbidity, and diagnostic uncertainty should remain distinct. Section 6.4 discusses the separate prospective association between childhood ADHD and later borderline personality disorder [36].

### 8.5. Sleep Disruption and Chronic Stress

Chronic sleep deprivation, whether from insomnia, an undiagnosed sleep-disordered breathing condition, shift work or simply overcommitment, degrades attention, working memory and impulse control in ways that are difficult to distinguish subjectively from ADHD, particularly because poor sleep and ADHD frequently co-occur and can aggravate one another. Prolonged occupational or caregiving stress produces a similar profile of fatigue-driven inattention and irritability. Adult ADHD assessment should therefore routinely screen for sleep quality and current life stressors, both because they are common alternative explanations for the presenting complaint and because, when they coexist with genuine ADHD, they require independent clinical attention rather than being subsumed entirely under a single diagnostic label. A meta-analysis of sleep in adults with ADHD found consistent differences from non-ADHD adults on subjectively reported sleep measures but few differences on objective, polysomnographic parameters, a pattern that itself illustrates how much of the overlap between ADHD and sleep disruption depends on self-report rather than independently verified findings [3,10,12,24].

Additional alternatives and comorbidities include autism-spectrum conditions, learning disorders, early neurocognitive decline, trauma-related disorders, medication effects, and general medical or sleep conditions affecting attention. These possibilities should be considered when the presentation is atypical, episodic, context-dependent, or fails to respond as expected (Table 2); the present narrative review does not estimate comparative misclassification frequencies across these conditions [3,10,12].

## 9. The Limits of Adult Self-Report Screening

Adult ADHD assessment commonly uses validated self-report screeners such as the Adult ADHD Self-Report Scale (ASRS) [6] and semi-structured diagnostic interviews such as the DIVA-5 [46]. These instruments serve different purposes: a screener identifies people who may require further evaluation, whereas a diagnostic interview structures a fuller clinical assessment and does not replace clinical judgement or collateral history [3,10,46]. Screening items such as difficulty concentrating, restlessness, disorganization, and forgetfulness are not specific to ADHD, particularly in people with anxiety, depression, poor sleep, or other psychiatric conditions [6,10,26].

These overlaps create an interpretive hazard in psychiatrically complex adults. A patient with prominent anxiety, depressive rumination, or sleep disruption can score above an ADHD screening cutoff without meeting full diagnostic criteria after developmental history and differential diagnosis are considered. Elevated scores should therefore prompt fuller evaluation and should be communicated as screening, rather than diagnostic, results [6,10,26,46].

## 10. Retrospective Diagnosis and the Problem of Childhood Recall

A defining feature of adult ADHD diagnosis is that it depends on establishing a childhood onset, yet the primary source of that evidence is usually the adult’s own recollection, filtered through decades of intervening experience. Mannuzza and colleagues demonstrated, in a landmark study, that adults with a well-documented history of childhood ADHD often failed to recall their own childhood symptoms accurately when interviewed later in life, with a general tendency toward underreporting rather than overreporting [7]. Subsequent work examining the concordance between self-report, informant report and the diagnostic method used to establish adult persistence has shown that estimated persistence rates vary substantially, in some analyses by an order of magnitude, depending on whether self-report or informant report is used and on how strictly DSM criteria are applied, underscoring how unstable any single retrospective account can be [8].

Studies comparing retrospective reports with prospectively collected childhood data show that recall of symptom onset is imperfect and cannot be assumed to err safely in one direction [7,8,13]. When school records or childhood informants are unavailable, current self-report should be interpreted alongside present mood, current symptom perception, functional history, and other available collateral evidence. The absence of records should not automatically exclude ADHD, but it should be documented as a source of uncertainty within a full developmental and differential assessment [3,10,12].

## 11. Occupational and Medico-Legal Dimensions Specific to ADHD

Adult ADHD evaluations may occur in clinical, academic, occupational, or forensic contexts. In the latter settings, the clinical diagnosis and eligibility for a specific accommodation or benefit are related but distinct questions, because administrative criteria are jurisdiction- and context-specific. The evaluator should document the evidence supporting the diagnosis and address functional impairment separately from the requested administrative outcome [10].

Stimulant treatment requires additional care because prescribed stimulants have recognized misuse and diversion potential [42]. When the history or assessment context raises a specific validity concern, clinicians should assess it using appropriate methods without presuming deceptive intent from help-seeking or medication interest alone. Symptom- and performance-validity measures can provide relevant evidence in neuropsychological evaluations, although their role differs from ordinary clinical diagnosis [47]. Treatment response should not be used to establish or retrospectively validate ADHD [3,10].

A later ADHD diagnosis may prompt reconsideration of an earlier formulation. Such reassessment should account for changes in diagnostic criteria, improved recognition of women and inattentive presentations, and the limitations of long-delayed recall [4,7,11,13]. A later diagnosis alone cannot determine whether the earlier assessment was unreasonable under the evidence and standards available at that time. Where the issue is clinically or legally relevant, the appropriate task is to compare the developmental, functional, collateral, and differential evidence supporting each formulation [3,10,12].

## 12. Toward a Disciplined Diagnostic Approach

The concerns raised in this review do not argue against the validity or clinical importance of ADHD. Persistent, developmentally rooted attentional and self-regulatory impairment is treatable, and improved adult recognition has benefited people whose ADHD was missed earlier [2,3,11,12]. The practical implication is that first-time adult assessment should document developmental onset and cross-situational impairment, seek collateral information where available, evaluate alternative and co-occurring conditions, and interpret screening scores within a full clinical assessment [3,4,10,12]. In academic, occupational, or forensic settings, the clinical conclusion should also be distinguished from the separate administrative or legal determination.

Table 3 translates the diagnostic standards, guideline and consensus recommendations, empirical evidence, and clinical reasoning discussed above into a practical framework. The new evidence/source and status column distinguishes (i) diagnostic standards; (ii) guideline, consensus, or empirical support; and (iii) the authors’ clinical synthesis. Items explicitly labelled as authors’ clinical synthesis are not presented as formally graded recommendations or as an established standard of care [3,4,10,12].

***Limitations.*** As a narrative rather than a systematic review, this article carries specific limitations that qualify its central arguments. The literature was identified through targeted searches of two databases rather than a fully reproducible, multi-database protocol; no formal risk-of-bias appraisal or independent dual screening was undertaken; and study selection reflected the authors’ clinical judgement rather than prespecified, independently applied inclusion criteria. The claim that adult ADHD faces a meaningful risk of diagnostic inflation should therefore be read as a clinically grounded hypothesis rather than as a claim directly demonstrated by diagnostic-validity data, population-level false-positive rates, or outcomes following independent reassessment. Rising diagnostic rates, criterial broadening and the well-documented unreliability of retrospective recall are compatible with this hypothesis; expanded telehealth access, by contrast, is direct evidence only of expanded diagnostic opportunity and detection, not of diagnostic inflation specifically, and it is listed here as context for the rise in diagnoses rather than as evidence for inflation itself. This narrative review did not identify population-scale evidence of that kind; its absence from the reviewed literature is one of the gaps highlighted here. Most of the literature informing this review also originates from the United States and a small number of high-income countries with comparable diagnostic systems and health-care access; a global meta-analysis of adult ADHD prevalence has documented substantial cross-national variation that is itself only partly explained by diagnostic methodology [48], so conclusions about the balance between underdiagnosis and overdiagnosis in this review may not generalize to jurisdictions with different diagnostic conventions, service structures or telehealth regulation, and this should be tested directly rather than assumed.

***Balancing underdiagnosis and overdiagnosis.*** The risks discussed here do not outweigh the well-documented harms of continued underdiagnosis, including academic and occupational impairment, comorbidity, relationship strain, and delayed access to treatment [2,3,11]. Nothing in this review should support diagnostic hesitancy toward patients, particularly women and people with predominantly inattentive presentations, for whom evidence of underrecognition is better established than evidence of overdiagnosis [11]. DSM-5 requires functional impairment across settings as well as symptom counts; evaluating impairment and the effect of compensatory supports is therefore more informative than symptom counts alone [4].

***Future research.*** Several questions raised throughout this review would benefit from focused empirical study rather than clinical reasoning alone: prospective, diagnostic-accuracy studies of first-time adult ADHD evaluations against independent, multi-informant reference standards; head-to-head comparisons of diagnostic quality and outcomes between telehealth and in-person assessment; validation of structured approaches to collateral-history reconstruction when school records or informants are unavailable; and longitudinal follow-up of apparent late-onset cases to determine how many represent true late emergence, subthreshold childhood presentations, or measurement artefact; and prospective studies quantifying how often adult ADHD is diagnosed in place of, rather than alongside, a better-fitting borderline personality disorder formulation, a question this review identifies on the basis of symptom overlap and longitudinal association but cannot quantify.

## 13. Conclusions

ADHD can be simultaneously valid, historically underrecognized, and vulnerable to over-attribution in a subset of adult assessments; the reviewed evidence does not quantify population-level false-positive diagnoses. Childhood onset, developmental change, and persistence are well established [1,2,3,19,20], while DSM-5 broadened adult eligibility in specific, measurable ways [4,9]. Many people benefit from a diagnosis made later in life [3,11]. However, the nonspecificity of screening symptoms and the limitations of retrospective recall mean that a first-time adult diagnosis should not rest on a screening score or current self-narrative alone [6,7,8,10,13]. This is a rationale for diagnostic safeguards, not evidence that diagnostic inflation has been demonstrated.

Maintaining diagnostic integrity requires holding several facts in view at once: many cases were genuinely missed; some apparent adult-onset presentations reflect subthreshold childhood traits or withdrawn environmental support; and current symptoms may also arise from alternative or co-occurring conditions. A defensible adult diagnosis therefore integrates developmental evidence, current symptoms, impairment across settings, collateral information where available, and systematic differential diagnosis [3,4,10,12,28]. Where evidence remains incomplete, the uncertainty should be documented rather than resolved by confidence alone.

## Figures and Tables

**Table 1:** Predominant clinical presentation of ADHD across three developmental windows. Evidence sources are cited within each row.

Developmental Stage	Predominant Presentation	Typical Informant(s)	Common Source of Under-Recognition
Early/middle childhood (~6–10 y)	Overt hyperactivity, impulsivity, motor restlessness [2,4,17]	Parents, teachers [3,4]	Quiet, inattentive-predominant, compliant children are overlooked [11,17]
Adolescence (~11–17 y)	Inner restlessness, organizational failure as scaffolding is withdrawn [2,3,19,20]	Self, parents, teachers [3]	Symptoms misattributed to motivation, mood, or normal adolescent variability [3,11]
Adulthood (18+ y)	Executive dysfunction: disorganization, procrastination, forgetfulness, impulsive decisions [2,3,4,18]	Self (often sole informant) [3,7,8,10]	Overlap with mood, anxiety, substance use, sleep and personality pathology [3,14,24,25,26,27]

**Table 2:** Key phenomenological discriminators between adult ADHD and its principal differential diagnoses. Sources are cited within rows.

Condition	Core Overlap with ADHD	Principal Discriminating Feature
Major depressive/bipolar-spectrum disorders	Poor concentration, indecision, impulsivity (hypomania)	Episodic course tied to mood state vs. trait-like, context-independent inattention since childhood [3,14,25,27]
Generalized anxiety disorder	Perceived inability to concentrate, feeling overwhelmed	Content-linked worry-driven distraction that resolves with the worry vs. content-free attentional lapse [3,25,26,27]
Substance use disorder	Attention/memory impairment, impulsivity	Temporal relationship to use/withdrawal; requires a period of abstinence to clarify [3,18,41,42]
Borderline personality disorder	Impulsivity, emotional reactivity, instability	Impulsivity tied to interpersonal/affective triggers vs. context-independent inhibitory deficit [14,15,16,43,44,45]
Sleep disruption/chronic stress	Inattention, forgetfulness, irritability	Improvement with corrected sleep/stress vs. persistence independent of sleep or stressor [3,12,24]

**Table 3:** Elements of a disciplined adult ADHD evaluation. The evidentiary status and source of each item are shown in a dedicated column.

Domain	Recommended Practice	Evidence/Source and Status
Developmental history	Obtain a full developmental and psychiatric history; seek collateral sources where available, and document their absence and the resulting uncertainty.	Diagnostic standard and guideline/consensus: DSM-5 [4]; European Consensus [3]; NICE NG87 [12]; Sibley [10].
Differential diagnosis	Assess alternative and co-occurring mood, anxiety, substance-use, sleep, stress-related, and personality conditions before attributing symptoms to ADHD.	Guideline/consensus: European Consensus [3]; NICE NG87 [12]; Sibley [10].
Screening instruments	Use validated screeners to identify the need for further evaluation, not to diagnose ADHD from a score alone; embed structured interviews within a full clinical assessment.	Screening/diagnostic evidence and guidance: ASRS [6]; DIVA-5 [46]; Sibley [10]; anxiety-sample validity study [26].
Late-onset presentations	Neither dismiss nor automatically accept; evaluate impairment, substance use, psychiatric explanations, subthreshold childhood traits, and recall limitations.	Prospective/reassessment evidence: Sibley et al. [28,35]; recall evidence [13].
Rediagnosis after a prior differing opinion	Reconstruct the available developmental and collateral evidence; a later diagnosis alone does not determine whether the earlier formulation was unreasonable.	Authors’ clinical synthesis based on recall evidence and diagnostic guidance [7,8,10,13]; not a formal standard of care.
Occupational/forensic context	Document the clinical diagnosis separately from jurisdiction-specific administrative eligibility or accommodation criteria.	Authors’ clinical synthesis informed by first-time diagnosis and validity guidance [10,47]; administrative requirements remain jurisdiction-specific.
Pharmacological treatment	When clinically indicated, assess misuse, diversion, or validity concerns without presuming deceptive intent; do not use treatment response to validate the diagnosis.	Guideline/evidence plus authors’ clinical synthesis: European Consensus [3]; NICE NG87 [12]; validity evidence [47]; misuse/diversion review [42].

## Data Availability

No new data were created or analyzed in this study. Data sharing is not applicable to this article.

## Abbreviations

The following abbreviations are used in this manuscript:

ADHD	attention-deficit/hyperactivity disorder
ASRS	Adult ADHD Self-Report Scale
DIVA-5	Diagnostic Interview for ADHD in Adults, 5th ed.
DSM-5	Diagnostic and Statistical Manual of Mental Disorders, 5th ed.
DSM-5-TR	DSM-5, Text Revision
GWAS	genome-wide association study
ICD-11	International Classification of Diseases, 11th Revision
MMWR	Morbidity and Mortality Weekly Report
WHO	World Health Organization

## References

[1] Biederman J., Petty C.R., Evans M., Small J., Faraone S.V. (2010). How persistent is ADHD? A controlled follow-up study of children with ADHD into early adulthood. Psychiatry Res..

[2] Faraone S.V., Asherson P., Banaschewski T., Biederman J., Buitelaar J.K., Ramos-Quiroga J.A., Rohde L.A., Sonuga-Barke E.J.S., Tannock R., Franke B. (2015). Attention-deficit hyperactivity disorder. Nat. Rev. Dis. Primers.

[3] Kooij J.J.S., Bijlenga D., Salerno L., Jaeschke R., Bitter I., Balázs J., Thome J., Dom G., Kasper S., Filipe C.N. (2019). Updated European Consensus Statement on diagnosis and treatment of adult ADHD. Eur. Psychiatry.

[4] American Psychiatric Association (2013). Diagnostic and Statistical Manual of Mental Disorders.

[5] Staley B.S., Robinson L.R., Claussen A.H., Katz S.M., Danielson M.L., Summers A.D., Farr S.L., Blumberg S.J., Tinker S.C. (2024). Attention-Deficit/Hyperactivity Disorder Diagnosis, Treatment, and Telehealth Use in Adults—National Center for Health Statistics Rapid Surveys System, United States, October–November 2023. MMWR Morb. Mortal. Wkly. Rep..

[6] Adler L.A., Spencer T., Faraone S.V., Kessler R.C., Howes M.J., Biederman J., Secnik K. (2006). Validity of pilot Adult ADHD Self-Report Scale (ASRS) to rate adult ADHD symptoms. Ann. Clin. Psychiatry.

[7] Mannuzza S., Klein R.G., Klein D.F., Bessler A., Shrout P. (2002). Accuracy of adult recall of childhood attention deficit hyperactivity disorder. Am. J. Psychiatry.

[8] Sibley M.H., Mitchell J.T., Becker S.P. (2016). Method of adult diagnosis influences estimated persistence of childhood ADHD: A systematic review of longitudinal studies. Lancet Psychiatry.

[9] Matte B., Anselmi L., Salum G.A., Kieling C., Gonçalves H., Menezes A., Grevet E.H., Rohde L.A. (2015). ADHD in DSM-5: A field trial in a large, representative sample of 18- to 19-year-old adults. Psychol. Med..

[10] Sibley M.H. (2021). Empirically-informed guidelines for first-time adult ADHD diagnosis. J. Clin. Exp. Neuropsychol..

[11] Attoe D.E., Climie E.A. (2023). Miss. Diagnosis: A systematic review of ADHD in adult women. J. Atten. Disord..

[12] National Institute for Health and Care Excellence (NICE) (2018). Attention Deficit Hyperactivity Disorder: Diagnosis and Management.

[13] Breda V., Rohde L.A., Menezes A.M.B., Anselmi L., Caye A., Rovaris D.L., Vitola E.S., Bau C.H.D., Grevet E.H. (2020). Revisiting ADHD age-of-onset in adults: To what extent should we rely on the recall of childhood symptoms?. Psychol. Med..

[14] Asherson P., Young A.H., Eich-Höchli D., Moran P., Porsdal V., Deberdt W. (2014). Differential diagnosis, comorbidity, and treatment of attention-deficit/hyperactivity disorder in relation to bipolar disorder or borderline personality disorder in adults. Curr. Med. Res. Opin..

[15] Weiner L., Perroud N., Weibel S. (2019). Attention deficit hyperactivity disorder and borderline personality disorder in adults: A review of their links and risks. Neuropsychiatr. Dis. Treat..

[16] MacDonald L., Sadek J. (2023). Management strategies for borderline personality disorder and bipolar disorder comorbidities in adults with ADHD: A narrative review. Brain Sci..

[17] Cabral M.D.I., Liu S., Soares N. (2020). Attention-deficit/hyperactivity disorder: Diagnostic criteria, epidemiology, risk factors and evaluation in youth. Transl. Pediatr..

[18] Asherson P., Buitelaar J., Faraone S.V., Rohde L.A. (2016). Adult attention-deficit hyperactivity disorder: Key conceptual issues. Lancet Psychiatry.

[19] Faraone S.V., Biederman J., Mick E. (2006). The age-dependent decline of attention deficit hyperactivity disorder: A meta-analysis of follow-up studies. Psychol. Med..

[20] Sibley M.H., Arnold L.E., Swanson J.M., Hechtman L.T., Kennedy T.M., Owens E., Molina B.S.G., Jensen P.S., Hinshaw S.P., Roy A. (2022). Variable patterns of remission from ADHD in the Multimodal Treatment Study of ADHD. Am. J. Psychiatry.

[21] Kessler R.C., Adler L., Barkley R., Biederman J., Conners C.K., Demler O., Faraone S.V., Greenhill L.L., Howes M.J., Secnik K. (2006). The prevalence and correlates of adult ADHD in the United States: Results from the National Comorbidity Survey Replication. Am. J. Psychiatry.

[22] Polanczyk G.V., Willcutt E.G., Salum G.A., Kieling C., Rohde L.A. (2014). ADHD prevalence estimates across three decades: An updated systematic review and meta-regression analysis. Int. J. Epidemiol..

[23] Popit S., Serod K., Locatelli I., Stuhec M. (2024). Prevalence of attention-deficit hyperactivity disorder (ADHD): Systematic review and meta-analysis. Eur. Psychiatry.

[24] Díaz-Román A., Mitchell R., Cortese S. (2018). Sleep in adults with ADHD: Systematic review and meta-analysis of subjective and objective studies. Neurosci. Biobehav. Rev..

[25] Fu X., Wu W., Wu Y., Liu X., Liang W., Wu R., Li Y. (2025). Adult ADHD and comorbid anxiety and depressive disorders: A review of etiology and treatment. Front. Psychiatry.

[26] Alarachi A., Merrifield C., Rowa K., McCabe R.E. (2024). Are we measuring ADHD or anxiety? Examining the factor structure and discriminant validity of the Adult ADHD Self-Report Scale in an adult anxiety disorder population. Assessment.

[27] Katzman M.A., Bilkey T.S., Chokka P.R., Fallu A., Klassen L.J. (2017). Adult ADHD and comorbid disorders: Clinical implications of a dimensional approach. BMC Psychiatry.

[28] Sibley M.H., Rohde L.A., Swanson J.M., Hechtman L.T., Molina B.S.G., Mitchell J.T., Arnold L.E., Caye A., Kennedy T.M., Roy A. (2018). Multimodal Treatment Study of Children with ADHD (MTA) Cooperative Group. Late-onset ADHD reconsidered with comprehensive repeated assessments between ages 10 and 25. Am. J. Psychiatry.

[29] American Psychiatric Association (2022). Diagnostic and Statistical Manual of Mental Disorders.

[30] World Health Organization (2024). Clinical Descriptions and Diagnostic Requirements for ICD-11 Mental, Behavioural and Neurodevelopmental Disorders.

[31] Herman B.K., Faraone S.V., Cutler A.J., Newcorn J.H., LaFrance E.M., Ripper Lewis M., Ruetsch C. (2025). Validity of an online assessment of attention-deficit/hyperactivity disorder among a real-world sample of adults seeking web-based mental health care. J. Clin. Psychiatry.

[32] Moffitt T.E., Houts R., Asherson P., Belsky D.W., Corcoran D.L., Hammerle M., Harrington H., Hogan S., Meier M.H., Polanczyk G.V. (2015). Is adult ADHD a childhood-onset neurodevelopmental disorder? Evidence from a four-decade longitudinal cohort study. Am. J. Psychiatry.

[33] Agnew-Blais J.C., Polanczyk G.V., Danese A., Wertz J., Moffitt T.E., Arseneault L. (2016). Evaluation of the persistence, remission, and emergence of attention-deficit/hyperactivity disorder in young adulthood. JAMA Psychiatry.

[34] Caye A., Rocha T.B.-M., Anselmi L., Murray J., Menezes A.M.B., Barros F.C., Gonçalves H., Wehrmeister F., Jensen C.M., Steinhausen H.-C. (2016). Attention-deficit/hyperactivity disorder trajectories from childhood to young adulthood: Evidence from a birth cohort supporting a late-onset syndrome. JAMA Psychiatry.

[35] Mitchell J.T., Sibley M.H., Hinshaw S.P., Kennedy T.M., Chronis-Tuscano A., Arnold L.E., Swanson J.M., Hechtman L.T., Molina B.S.G., Caye A. (2021). A qualitative analysis of contextual factors relevant to suspected late-onset ADHD. J. Atten. Disord..

[36] O’Grady S.M., Hinshaw S.P. (2023). Developmental predictors of young adult borderline personality disorder: A prospective, longitudinal study of females with and without childhood ADHD. BMC Psychiatry.

[37] Brancati G.E., Perugi G., Milone A., Masi G., Sesso G. (2021). Development of bipolar disorder in patients with attention-deficit/hyperactivity disorder: A systematic review and meta-analysis of prospective studies. J. Affect. Disord..

[38] Barkley R.A. (1997). Behavioral inhibition, sustained attention, and executive functions: Constructing a unifying theory of ADHD. Psychol. Bull..

[39] Volkow N.D., Wang G.J., Kollins S.H., Wigal T.L., Newcorn J.H., Telang F., Fowler J.S., Zhu W., Logan J., Ma Y. (2009). Evaluating dopamine reward pathway in ADHD: Clinical implications. JAMA.

[40] Demontis D., Walters G.B., Athanasiadis G., Walters R., Therrien K., Nielsen T.T., Farajzadeh L., Voloudakis G., Bendl J., Zeng B. (2023). Genome-wide analyses of ADHD identify 27 risk loci, refine the genetic architecture and implicate several cognitive domains. Nat. Genet..

[41] Wilens T.E., Martelon M., Joshi G., Bateman C., Fried R., Petty C., Biederman J. (2011). Does ADHD predict substance-use disorders? A 10-year follow-up study of young adults with ADHD. J. Am. Acad. Child Adolesc. Psychiatry.

[42] Wilens T.E., Adler L.A., Adams J., Sgambati S., Rotrosen J., Sawtelle R., Utzinger L., Fusillo S. (2008). Misuse and diversion of stimulants prescribed for ADHD: A systematic review of the literature. J. Am. Acad. Child Adolesc. Psychiatry.

[43] Gunderson J.G., Herpertz S.C., Skodol A.E., Torgersen S., Zanarini M.C. (2018). Borderline personality disorder. Nat. Rev. Dis. Primers.

[44] Sharp C., Fonagy P. (2015). Practitioner review: Borderline personality disorder in adolescence. J. Child Psychol. Psychiatry.

[45] Philipsen A., Limberger M.F., Lieb K., Feige B., Kleindienst N., Ebner-Priemer U., Barth J., Schmahl C., Bohus M. (2008). Attention-deficit hyperactivity disorder as a potentially aggravating factor in borderline personality disorder. Br. J. Psychiatry.

[46] Di Lorenzo R., Latella E., Gualtieri F., Adriani A., Ferri P., Filippini T. (2025). Validity of the Italian Version of DIVA-5: Semi-Structured Diagnostic Interview for Adult ADHD Based on the DSM-5 Criteria. Healthcare.

[47] White D.J., Ovsiew G.P., Rhoads T., Resch Z.J., Lee M., Oh A.J., Soble J.R. (2022). The divergent roles of symptom and performance validity in the assessment of ADHD. J. Atten. Disord..

[48] Song P., Zha M., Yang Q., Zhang Y., Li X., Rudan I. (2021). The prevalence of adult attention-deficit hyperactivity disorder: A global systematic review and meta-analysis. J. Glob. Health.

